# Effect of Psychological Capital of Volunteers on Volunteering Behavior: The Chained Mediation Role of Perceived Social Support and Volunteer Motivation

**DOI:** 10.3389/fpsyg.2021.657877

**Published:** 2021-09-17

**Authors:** Li ping Xu, Jin bao Liao, Yu shen Wu, Hong da Kuang

**Affiliations:** ^1^Department of Social Science, Zhuhai of Zunyi Medical University, Zhuhai, China; ^2^Guangdong Communication Polytechnic, Guangzhou, China; ^3^Faculty of Psychology, Beijing Normal University, Beijing, China; ^4^School of Marxism, Guilin University of Electronic Technology, Guilin, China

**Keywords:** volunteer, psychological capital, perceived social support, voluntary motivation, volunteering behavior

## Abstract

This study explored the role of perceived social support and voluntary motivation in the effect of psychological capital of volunteers on volunteering behavior. A sample of 1,165 volunteers who were registered in the China Voluntary Service Information System was investigated using a self-reported questionnaire, showing that the psychological capital, perceived social support, voluntary motivation, and volunteering behavior of the volunteers were significantly and positively related to each other. The psychological capital of the volunteers affected volunteering behavior not only directly, but also indirectly through the mediating role of voluntary motivation. Moreover, perceived social support and voluntary motivation also played a chain role in the relationship between the psychological capital and volunteering behavior of the volunteers. Therefore, increasing the psychological capital of the volunteers should promote their perceived social support and inspire voluntary motivation, in turn affecting their volunteering behavior.

## Introduction

Volunteers are defined as members of organized groups who engage in social public service activities without remuneration or publicity. Musick and Wilson (2003) believe that the scale and service effectiveness of volunteer organizations can be regarded as a barometer of social health. In fact, volunteering behavior not only holds great economic benefit for a country, but also brings warmth, love, and well-being to the people (Stukas et al., 2016). Previous studies have shown that the effectiveness of volunteer service is often closely related to the positive mental quality of the volunteers (Li and Zhou, 2017; Xu et al., 2020). Actually, to promote and strengthen volunteer service, it is essential to explore the positive mental quality and volunteering behavior of the volunteers. Moreover, the core element of promoting and strengthening voluntary service is the mental health of people, especially the mental state and quality of volunteers, which plays an important role in the generation and development of their volunteering behaviors, and the core content of the mental state and quality of volunteers is the psychological capital of volunteers. Most studies of voluntary service have focused on the macro level of volunteer function and motivation (Pauline and Pauline, 2009; Arbak and Villeval, 2013; Dickson et al., 2013, 2015; Li L. et al., 2016), voluntary management and incentive (Prestby et al., 1990; King, 2018; Gong and Li, 2019), values and volunteerism (Johnson et al., 1998; Song et al., 2016), and leisure perspectives (Green and Chalip, 2004). Moreover, the research on volunteering behavior at present also focuses on physical, social, and cultural capital (Xu et al., 2020). However, previous research on the psychological analysis of the mechanism, whereby they influence volunteering behavior, has been relatively rare. Moreover, there have been very few studies on volunteering behavior from the perspective of psychological capital. Studies have reported that psychological capital goes far beyond physical, social, and cultural capital, showing how the positive psychological state in individual development affects individual cognition and behavior, and brings unexpected help to work and life of the individual (Luthans and Youssef, 2004; Luthans et al., 2007).

According to the conservation of resources theory, positive psychological quality, helpful social support, and contextual factors are considered to be valuable resources (Hobfoll and London, 1986; Hobfoll, 1989), and the tendency of individuals to try to conserve resources is one of the key factors to explain changes in individual psychology and behavior (Halbesleben et al., 2014). Individuals with abundant resources may have more enthusiasm and energy to drive them to have more positive behaviors (Hobfoll, 2011; Hobfoll et al., 2018). Moreover, previous studies have found that perceived social support plays an important role in protection from depression, releasing work-related stress, improving mental health, and enhancing well-being (Guerette and Smedema, 2011; Gariépy et al., 2016; Chen et al., 2020; Jung and Baek, 2020; Lecca et al., 2020). These studies provided a theoretical reference to reveal the impact of perceived social support on voluntary behavior. Additionally, self-determination theory holds that the causal orientation of an individual and social environment work together to promote the internalization of internal motivation and external motivation by satisfying the three psychological needs of an individual, namely: autonomy, relationship, and competence, and ultimately change the behavior of the individual (Deci and Ryan, 2000). This provides a theoretical support for revealing the influence of voluntary motivation on voluntary behavior.

Therefore, in the process of the occurrence, development, and formation of volunteering behavior, changes in mental quality, emotion, and cognition of volunteers caused by psychological resources such as psychological capital, voluntary motivation, and perceived social support, might be more closely related to volunteering behavior. However, how does the psychological capital of volunteers affect their volunteering behavior? What is the mechanism? These questions await further research. Although Li and Zhou (2017) mentioned a relationship between psychological capital (PsyCap) and altruistic behavior, the dimensions of their psychological capital follow the structure of an ordinary individual psychological capital. Therefore, based on conservation of resources theory and self-determination theory, this study introduces two elements of perceived social support and voluntary motivation to explore the mediating path between them, thus revealing the “black box” of the influence of psychological capital of volunteers on the volunteering behavior and its mechanism, which provides a new theoretical perspective to enrich the application of psychological capital, while broadening research on the sustainable development of volunteering behavior.

## Theory and Hypothesis

### The Positive Predictive Effect of the Psychological Capital of Volunteers on Volunteering Behavior

Psychological capital, as a comprehensive mental resource, is a positive mental quality and state in the process of individual growth and development that can promote positive cognition, attitude, and behavior of an individual (Luthans et al., 2004). Meanwhile, self-efficacy, optimism, hope, and resilience are considered the structural dimensions of general individual psychological capital (Luthans and Youssef, 2004; Luthans et al., 2007). The conservation of resources theory holds that psychological capital is also a type of psychological resource that helps to strengthen more positive behaviors (Hobfoll et al., 2018; Xu et al., 2020). However, there have been few reports on research in the psychological capital of volunteers. As a special group, volunteers have distinctive characteristics regarding the structural dimension of their psychological capital. In a previous study, we stated that the psychological capital of volunteers, which refers to the positive mental quality or state of volunteers in the process of volunteering, determines whether individuals continue to participate in voluntary activities and perform volunteering effectively; it includes the five dimensions of self-efficacy, sense of responsibility, gratitude, resilience, and hope (Xu and Han, 2020). In addition, there have been very few reports on the relationship between the psychological capital of volunteers and volunteering behavior in the past. However, research on the relationship between psychological capital and altruistic behavior (Gholampour and Mohammad, 2017; Li and Zhou, 2017), service behavior (Cheng et al., 2018), and organizational citizenship behavior (Avey et al., 2010; Harms and Luthans, 2012) has shown that high-level psychological capital is significant for the emergence and development of altruistic behavior, service behavior, and organizational citizenship behavior. These studies provide a theoretical reference for how the psychological capital of volunteers can effectively stimulate or promote the generation, stability, and development of volunteering behavior. Thus, this study proposes that the PsyCap of volunteers would have a positive predictive effect on volunteering behavior (Hypothesis 1).

### Mediating the Effect of Perceived Social Support of the Volunteers

Perceived social support emphasizes the social support of self-understanding and self-feeling of individuals, which refers to three dimensions including perceived social support from family, friends, and others (Blumenthal et al., 1987). It is not imaginary but a perception of objective support from the external environment (Thoits, 1983), and it is an important aspect of social support (Blumenthal et al., 1987). Besides, as an element of individual cognition, perceived social support is also the cognitive assessment of an individual that reflects a reliable relationship with others (Barrera, 1986). The theory of positive psychology posits that psychological capital is a psychological state or quality that can promote the positive cognition and action tendencies of individuals (Luthans et al., 2007; Avey et al., 2011; Zhu and Wang, 2011). Previous studies have found that the psychological capital of college students has a positive predictive effect on perceived social support (Jiankun et al., 2018). Besides, psychological capital, as a positive psychological quality or state, is usually manifested as positive cognition, rational attribution, strong antifrustration ability, and so on (Luthans et al., 2004), and can thus enhance the strong perception of support from friends, family, and society of the individuals (Jiankun et al., 2018). Moreover, perceived social support as a subjective support may be more meaningful than actual support. Because although subjective support is not objective reality, “the perceived reality is the psychological reality, and it is the psychological reality as the actual (mediating variable) variable that affects human behavior and development” (Thoits, 1983). In other words, perceived social support can also effectively predict individuals' behavior. Meanwhile, according to the theory of resource preservation, perceived social support is considered as another psychological resource, which can promote the occurrence of individual behavior (Hobfoll, 1989). It can be seen from the above that improving the psychological capital of volunteers helps to enhance the perceived social support of individuals, thereby contributing to the development of individual volunteer behavior. Taken together, based on these findings, this study proposes that perceived social support would play a mediating role on the impact of psychological capital on the volunteering behavior of volunteers (Hypothesis 2).

### Mediating Effect of Voluntary Motivation of Volunteers

Voluntary motivation is linked to an internal psychological process and behavioral dynamics that motivates individuals to participate in voluntary service, and the maintenance of sustainable volunteering behavior (Omoto and Snyder, 1995). In fact, volunteering behavior is also an external manifestation of voluntary motivation (Compton et al., 2004). Previous research has discussed the relationship between voluntary motivation and volunteering behavior more theoretically, resulting in the self-determination theory (Deci and Ryan, 1985), trait model theory (Carlo et al., 2005), the motivational behavior theory (Snyder and Omoto, 2008), system quality of life theory (Shye, 2010), and integrated model theory (Penner, 2010). These theories would reveal the predictive effects of voluntary motivation on the generation, development, and sustainability of volunteering behavior from different perspectives. For example, self-determination theory is the motivation theory of human behavior, which reveals the effective path of external intervention affecting individual motivation, and expounds the process of an external environment promoting internal motivation and the internalization of external motivation and thus changes behavior (Deci and Ryan, 1985). Meanwhile, this intrinsic motivation can help individuals to promote the activation, orientation, maintenance, and development of volunteering behavior (Scheuthle et al., 2005; Snyder and Omoto, 2008; Li C. et al., 2016). Clary et al. (1998) believe that the motivation for individual volunteering behavior includes six aspects, which are as follows: improving the expression of values, professional skills, social interaction, self-enhancement, self-protection, and knowledge understanding. Empirical studies have also confirmed the positive predictive effect of voluntary motivation on volunteering behavior (Shipway et al., 2012; Dickson et al., 2015; Okun et al., 2015). Moreover, psychological capital has a stimulating effect on the improvement of intrinsic motivation of individuals (Siu et al., 2013; Datu et al., 2016). In addition, a study has shown that there are mediating effects of intrinsic motivation in relationships between psychological capital and in-role behavior among cosmetology workers (Kwon, 2018), and another study has shown that there is a mediating effect of internal motivation on the relationship between psychological capital and innovation behavior (Quan and Shen, 2017). Thus, this study also proposes that voluntary motivation would play a mediating role in the impact of the psychological capital on the volunteering behavior of the volunteers (Hypothesis 3).

### The Chained Mediating Effect of Perceived Social Support and Voluntary Motivation of Volunteers

This study proposes the mediating effects of perceived social support and voluntary motivation in the relationship between psychological capital and voluntary behavior, respectively. The psychological capital of volunteers, as a positive psychological quality, can be regarded as a mental resource that has predictive effects on individual cognition, attitude, and behavior (Luthans et al., 2007), which can enhance individuals strong perception of support from others (Jiankun et al., 2018). Additionally, according to the conservation of resources theory, resource is defined as that which the individual perceives as being something that helps him achieve his goal (Halbesleben et al., 2014). This definition emphasizes the subjective perception and evaluation of whether specific things are helpful to achieve their goals, and emphasizes that the value of a specific resource depends on the degree to which it matches the current needs or goals of an individual (Halbesleben et al., 2014; Hobfoll et al., 2018). Therefore, perceived social support, as a positive cognition, is influenced by psychological capital (Luthans et al., 2007). Meanwhile, perceived social support is also regarded as a psychological resource that can help individuals to achieve their goals (Halbesleben et al., 2014), which can stimulate the motivation of volunteers, and thus promote the development of volunteer behavior (Omoto and Snyder, 1995). Moreover, previous studies have found the mediating effect of perceived social support in self-efficacy and achievement motivation in secondary vocational students (Ya-Ru et al., 2019). Studies have also found a relationship between the perceived social support and learning motivation in college students, and revealed the predictive effect of the perceived social support of family, friends, and other important people on the motivation for learning (Tezci et al., 2015). Besides, Horowitz et al. (2001) revealed that an individual with a clear goal actively seeking help is likely to obtain positive results in a good psychological state. This shows that perceived social support has significant effects on behaviors through psychological motivation within individuals. For the above-mentioned reasons, this study proposes that the perceived social support and voluntary motivation of volunteers would play a chained mediating role with the same impact that the psychological capital has on volunteering behavior of the volunteers (Hypothesis 4).

### The Present Study

**Figure 1:**
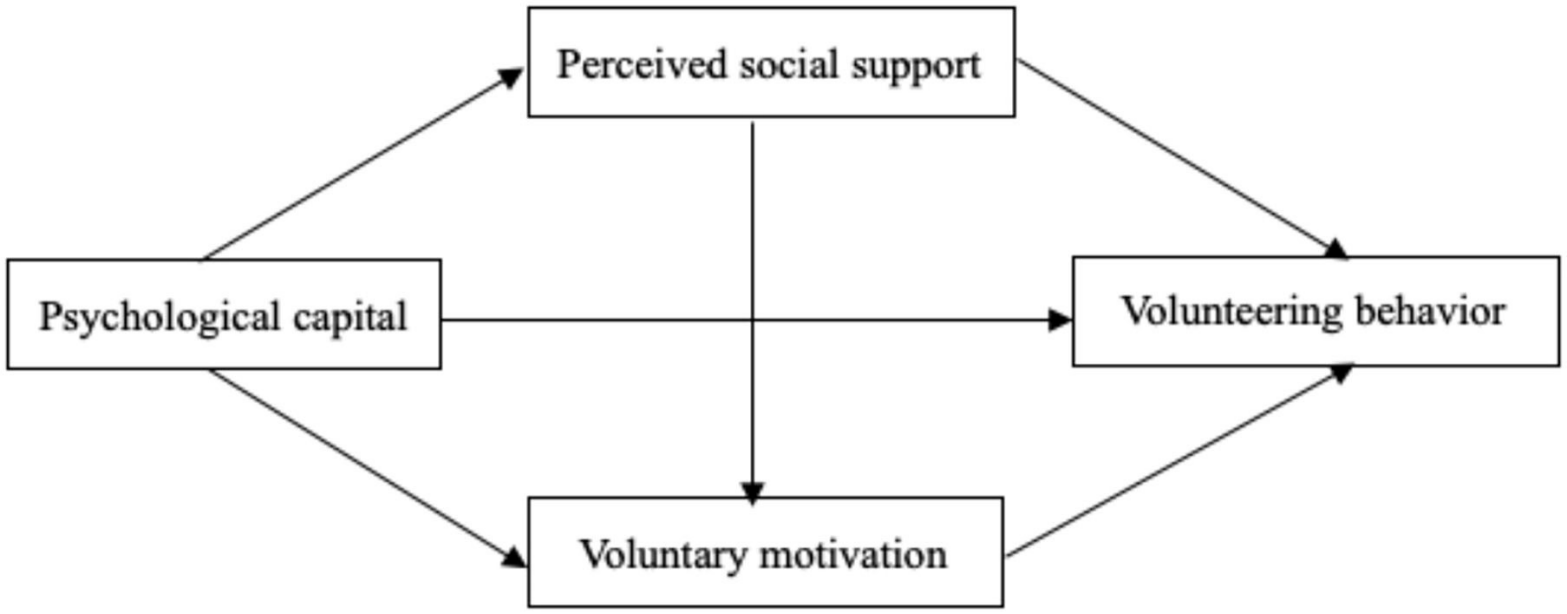
Model of the chained mediating effect of perceived social support and voluntary motivation.

The present study focused on the relationship between the psychological capital and volunteering behavior of the volunteers as well as the chained mediating effect of perceived social support and voluntary motivation. Taken together, based on theory and the hypotheses of the present study, a theoretical model of the chained mediating effect of perceived social support and voluntary motivation was constructed, as shown in Figure 1.

## Materials and Methods

### Participants and Procedure

This study was approved by six organizations of social workers and seven universities including Guangdong Teachers College of Foreign Language and Arts, Southern Medical University, Yichun University, Linyi University, Zhuhai Campus of Zunyi Medical University, Guangdong Polytechnic of Industry and Commerce, and the South China University of Technology, which are located in Guangdong, Jiangxi, Shandong, and Guizhou Provinces and in Shanghai, China. My team members and I contacted the leaders of voluntary organization within each unit and randomly distributed about 100–200 questionnaires with their help. All the respondents had volunteer service experience and were taking part in volunteer service training or volunteer work at that time, and the volunteers were recruited by the recruitment notice published on the internet and signed up voluntarily. Participants completed the survey individually on-site after providing their informed consent, all of them were volunteers without any compensation, and the data they filled out were completely anonymous.

A total of 1,600 questionnaires were distributed and 1,204 were collected, and we finally obtained 1,165 valid questionnaires after eliminating 39 questionnaires with incomplete information, with a questionnaire effective rate of 96.8%. The inclusion criterion for the participants was active registration in the CVSIS (China Voluntary Service Information System). All participants served in the community, such as maintaining community order and environment, helping orphans and school-age children, and promoting health knowledge. Participants were 60.4% females (704) and 39.6% males (461), in the age range 16–68 years. In terms of education subjects, there were 19.1% in the liberal arts (223), 25.7% in science and engineering (299), 22.2% in medical science (259), 9.7% in management (113), 4.5% in art (53), and 18.7% in others (218). In addition, 3.4% were civil servants (40), 51.8% college students (603), 16.7% freelancers (195), 20.5% personnel of enterprises and institutions (239), and 7.6% retirees (88).

### Measures

#### Psychological Capital of Volunteers

This questionnaire, based on the psychological capital questionnaire, was developed by Zhang et al. (2010) in accordance with the theory of Luthans et al. (2007). Meanwhile, following the preliminary results of grounded theory (Xu and Han, 2020), four items on responsibility mission (Xu and Li, 2019) and six items on gratitude (Mccullough et al., 2001) were added. Following the basic principle that the data of the exploratory factor analysis and the confirmatory factor analysis of the revised questionnaire cannot overlap or repeat, 552 questionnaires (47.4%) were randomly selected from the total sample for exploratory factor analysis. Confirmatory factor analysis was applied to the remaining 613 questionnaires (52.6%).

This questionnaire includes five dimensions as follows: sense of responsibility, gratitude, self-efficacy, resilience, and hope. The items with loadings <0.3 were deleted, and each dimension finally retained four items. The questionnaire comprises 20 items scored on a 7-point Likert scale ranging from 1 (very strongly disagree) to 7 (very strongly agree). Sense of responsibility refers to a positive psychological quality of the sense of responsibility of an individual in the pursuit of social values and social responsibilities. This dimension contains four items, such as “It is incumbent upon us to provide help to vulnerable groups.” Gratitude refers to the kind of compassion and positive mental state of individuals after receiving the favor of altruistic behavior. This dimension contains four items, such as “I am grateful for being given many wonderful things.” Self-efficacy refers to the self-consciousness of the individual that he or she is capable of accomplishing tasks and achieving success. This dimension contains four items, such as “I'm more capable than the average person.” Resilience refers to the ability of individual to recover quickly from adversity, frustration, and failure. This dimension contains four items, such as “I can quickly recover from the difficulties.” Hope refers to the positive state of achieving the predetermined goal in various ways. This dimension contains four items, such as “I think life is always good.” We calculated an average score for each item; the higher the score for each dimension, the higher the level of each factor. The structural validity of the scale was adequate (χ^2^/*df* = 3.743; NFI = 0.858; IFI = 0.892; TLI = 0.874; CFI = 0.891; RMSEA = 0.067). The reliability of this questionnaire is 0.857 in this study.

#### Perceived Social Support

This study used the perceived social support scale compiled by Blumenthal et al. (1987), and it was translated into Chinese by Jiang et al. (2006). Perceived social support refers to the extent to which an individual perceives social support from family, society, friends, and others. This scale used a 7-point Likert scale and comprised 12 items such as, “My family can help me concretely.” (1 = totally disagree, 7 = totally agree). We calculated the average score for each item. The higher the average score, the higher the perceived level of social support from others. The reliability of this scale is 0.897 in this study.

#### Voluntary Motivation

This study used the volunteer function motivation inventory compiled by Clary et al. (1998) and revised by Law et al. (2011) at the Chinese University of Hong Kong, which includes six dimensions, such as: understanding function motivation, enhancement function motivation, protective function motivation, values of function motivation, social function motivation, and career function motivation. This questionnaire consisted of 30 items scored on a 7-point Likert scale ranging from 1 (totally disagree) to 7 (totally agree). Understanding function motivation refers to participating in voluntary activities designed to gain new knowledge and exercise skills. This dimension contains five items, such as “Experience in volunteer activities may be the first step as I like to work.” Enhancement function motivation refers to seeking psychological growth and development, such as experiencing self-worth and enhancing self-esteem. This dimension contains five items, such as “Volunteer activities improved my self-esteem.” Protective function motivation refers to alleviating negative emotions by participating in volunteer work. This dimension contains five items, such as “I no longer feel lonely after participating in volunteer activities.” Values function motivation refers to expressing or practicing personal values such as humanitarianism and altruism. This dimension contains five items, such as “Volunteering makes me feel important.” Social function motivation refers to strengthening social connections. This dimension contains five items, such as “Volunteer activities are a way to make new friends.” Career function motivation refers to gaining career-related experiences. This dimension contains five items, such as “Volunteer activities provide me with opportunities to explore different careers.” We calculated the average score for each item. The higher score for each dimension indicates that the individual perceives a higher level of voluntary motivation. The reliability of this questionnaire is 0.956 in this study.

#### Volunteering Behavior

The present study used the volunteering behavior questionnaire developed by Carlo et al. (2005). This questionnaire comprises four items. Volunteers were asked whether they had ever volunteered (yes = 1, no = 0), are currently volunteering (yes = 1, no = 0), or planed to volunteer during the next 2 months (yes = 1, no = 0), and the likelihood that they would volunteer in the campus-based community service program if asked (definitely yes = 4, probably yes = 3, maybe = 2, probably no = 1, and definitely no = 0). We calculated an average score for each item by dividing the total score by 4. The final score of this questionnaire varied from 0 to 1.75, where a higher average score denotes a higher tendency to participate in volunteering. The reliability of this questionnaire is 0.762 in this study.

### Statistical Analyses

The present study used SPSS24.0 to calculate Cronbach's alpha coefficients, descriptive statistics, and interitem correlations analyses on the 1,165 questionnaires, and adopted Model 6 of the PROCESS procedure for SPSS24.0 (the model assumes that two variables have a chained mediating effect in the relationship between the independent and dependent variables, which is in coincidence with the theoretical model in this study) to carry out the bootstrap inspection of the 95% confidence interval of the mediating effect of perceived social support and voluntary motivation on the effect of the psychological capital of volunteers on volunteering behavior and setting the self-sampling number to 5,000 (Hayes, 2013).

## Results

### Common Method Deviation Analysis

Haman single-factor analysis method was used to analyze the common deviation method (CMD) of all the valid data in this study. The results showed that the featuring root value of 11 factors was >1, where the variance of the first was 14.956%, which was smaller than the critical value of 40%. The present study also carried out a confirmatory analysis of the single factor model, and the results showed that the model had fit badly (RMSEA = 0.19, χ^2^/*df* = 42.151, CFI = 0.63, TLI = 0.57), which meant that the CMD of this study was not remarkable.

### Preliminary Analysis

The results of the descriptive statistics and correlation analysis are shown in Table 1. The results revealed that the psychological capital of the volunteers had a positive correlation with factors such as volunteering behavior, voluntary motivation, and perceived social support. The correlation coefficient was 0.281–0.651 (*p* < 0.01), which showed that it was necessary to further reveal the internal relationship between the elements.

**Table 1:** Means, standard deviations and correlation coefficients of variables.

	***M***	***SD***	**1**	**2**	**3**
1. Psychological capital	4.89	0.67	—		
2. Perceived social support	5.59	0.92	0.544^**^	—	
3. Voluntary motivation	5.39	0.84	0.651^**^	0.606^**^	—
4. Volunteering behavior	1.38	0.41	0.461^**^	0.281^**^	0.384^**^

* *Represents p < 0.05*;

** *Represents p < 0.01. The same is as following*.

### Hypothesis Testing

Previous studies have shown that age, education and profession are important factors that influence volunteering behavior (Musick and Wilson, 2003; Haski-Leventhal et al., 2008) and that women are more likely to volunteer than men (Moore et al., 2014). Therefore, the present study viewed gender, age, education, and profession as the control variables when analyzing the mediating model of joint regulation in the relationship between the psychological capital and volunteering behavior of volunteers. In addition, the study applied centralization treatment to the variable data to avoid multicollinearity between the variables. On this basis, the present study adopted Model 6 of the PROCESS procedure for SPSS24.0 to explore the role of perceived social support and volunteer motivation in the relationship between the psychological capital and volunteering behavior of the volunteers.

The results showed that the total effect of volunteer psychological capital on volunteering behavior was significant (ß = 0.256, *p* < 0.01) and can positively predict volunteering behavior (ß = 0.21, *p* < 0.01); Hypothesis 1 was thus accepted. Moreover, psychological capital of the volunteers can significantly predict not only perceived social support (ß = 0.79, *p* < 0.01), but also voluntary motivation (ß = 0.55, *p* < 0.01). Moreover, voluntary motivation can significantly positively predict volunteering behavior (ß = 0.06, *p* < 0.01) (see Table 2).

**Table 2:** Analysis of chained mediation model.

**Regression equation**		**Index of overall fit**			**Significance of regression coefficient**			
**Result variable**	**Prediction variable**	***R***	***R^**2**^***	***F***	**ß**	**LLCI**	**ULCI**	***t***
Volunteering behavior		0.50	0.25	75.847^**^				
	Gender				0.012	−0.030	0.054	0.56
	Age				0.075	0.042	0.107	4.46^**^
	Profession				0.001	−0.029	0.026	0.03
	education subject				−0.020	−0.047	0.008	−1.40
	psychological capital				0.256	0.223	0.289	15.34^**^
Perceived social support		0.56	0.32	106.74^**^				
	Gender				0.17	0.077	0.258	3.63^**^
	Age				0.14	0.068	0.208	3.85^**^
	Profession				−0.10	−0.152	−0.049	−3.84^**^
	education subject				0.03	−0.037	0.081	0.73
	psychological capital				0.785	0.715	0.855	21.97^**^
Voluntary motivation		0.72	0.52	209.85^**^				
	Gender				0.01	−0.057	0.081	0.34
	Age				0.04	−0.016	0.092	1.37
	Profession				0.01	−0.034	0.044	0.25
	education subject				−0.06	−0.100	−0.010	−2.40^*^
	perceived social support				0.327	0.293	0.371	14.62^**^
	psychological capital				0.553	0.489	0.616	17.06^**^
Volunteering behavior		0.51	0.25	56.54^**^				
	Gender				0.01	−0.038	0.051	0.39
	Age				0.07	−0.016	0.092	4.17^**^
	Profession				0.002	−0.022	0.026	0.15
	education subject				−0.02	−0.044	−0.011	−1.12
	perceived social support				−0.003	−0.032	0.027	−0.17
	voluntary motivation				0.060	0.025	0.096	3.37^**^
	psychological capital				0.209	0.166	0.253	9.46^**^

* *p < 0.05*;

** *p < 0.01*.

**Figure 2:**
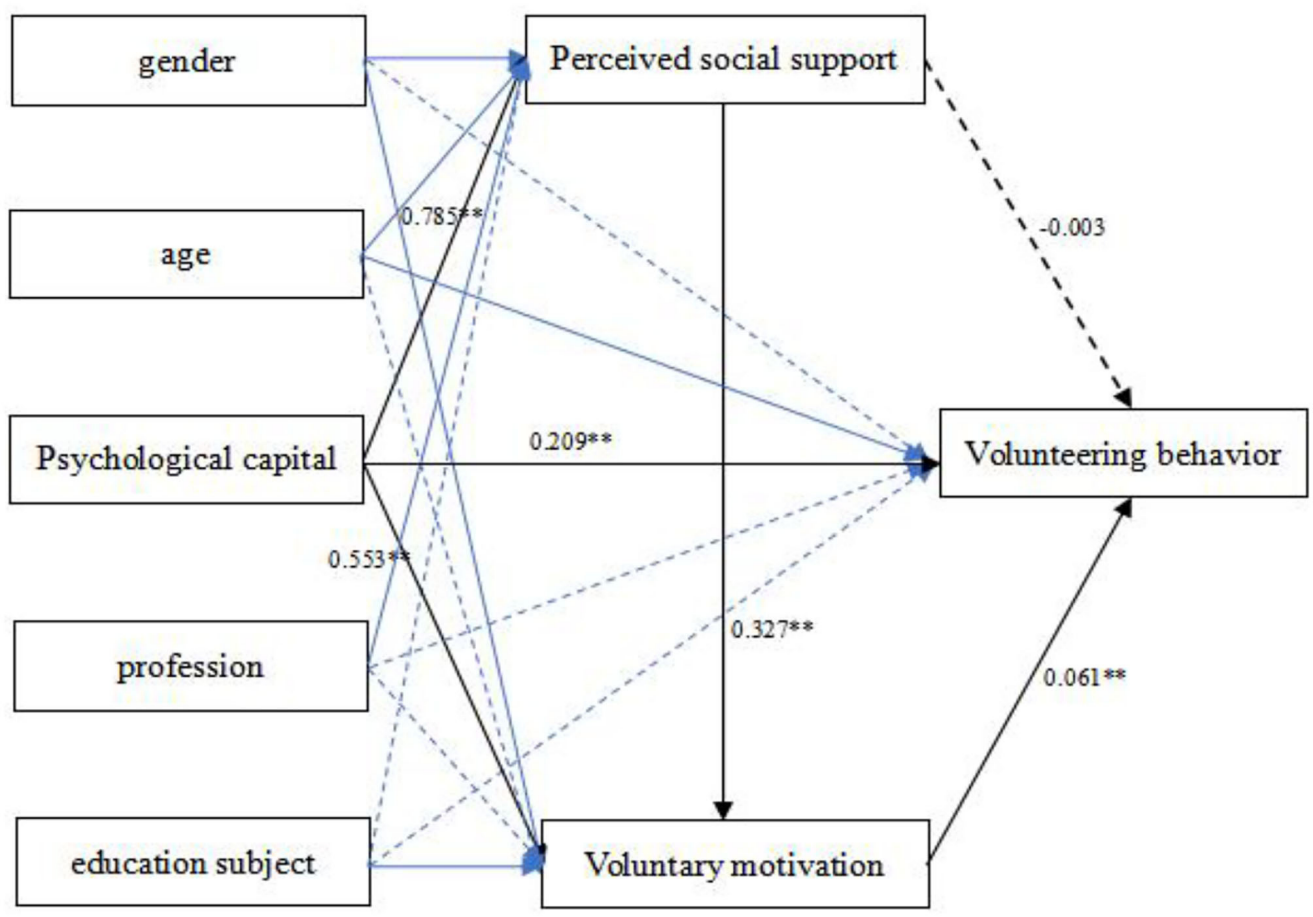
Chained mediation diagram. The solid line in the influence of demographic variables on each factor indicates *p* < 0.05; the dashed line indicates *p* > 0.05. The numbers in the figure indicate the path coefficients. *p < 0.05; **p < 0.01.

The result for the mediation effect showed (see Table 3 and Figure 2) that the bootstrap 95% confidence interval for the mediating effects of perceived social support and voluntary motivation was from 0.166 to 0.253, excluding 0, indicating that perceived social support and voluntary motivation were the mediating variables for the psychological capital of volunteers to influence volunteering behavior, which accounted for 18.4% of the total effect (effect value = 0.256), whereas the direct effect of volunteer psychological capital on volunteering behavior accounted for 81.6% of the total effect; that the indirect effect 1 (effect value = −0.002) was not significant for the path of the mediating effect of psychological capital → perceived social support → volunteering behavior (Hypothesis 2 was rejected); and that the mediating effects of perceived social support and voluntary motivation on the effect of volunteer psychological capital on volunteering behavior were mainly exerted through two paths, namely: indirect effect 2 (effect value = 0.016) mediated psychological capital → voluntary motivation → volunteering behavior (Hypothesis 3 was accepted), and indirect effect 3 (effect value = 0.033) was the chained mediating effect of psychological capital → perceived social support → voluntary motivation → volunteering behavior (Hypothesis 4 was accepted); and that indirect effects 2 and 3 accounted for 6.1% and 13.1% of the total effect, respectively.

**Table 3:** Test of mediation effect.

	**Indirect effect value**	**Boot standard error**	**Boot LLCI**	**Boot ULCI**	**Relative mediation effect%**
Total indirect effect	0.047	0.015	0.018	0.077	18.4
Indirect effect 1	−0.002	0.011	−0.023	0.202	−0.8
Indirect effect 2	0.016	0.006	0.005	0.027	6.1
Indirect effect 3	0.033	0.012	0.011	0.057	13.1

## Discussion

Correlation analyses reveal that the volunteering behavior of the volunteers has a significant positive correlation with psychological capital, voluntary motivation, and perceived social support, consistent with former studies on the relationships among psychological capital, volunteering behavior (Li and Zhou, 2017; Xu and Han, 2020), motivation (Siu et al., 2013; Ephrem et al., 2021), and perceived social support (Ren and Ji, 2019; Xu et al., 2020). This means that volunteers with higher levels of psychological capital tend to show a higher level of volunteering behavior, perceived social support, and voluntary motivation. Psychological capital is a positive psychological quality or state of an individual in the process of carrying out voluntary activities, and it is the source of motivation for individuals to continue participating in voluntary activities and effectively carry out volunteering behavior (Li and Zhou, 2017; Xu and Han, 2020). Besides, individuals with higher psychological capital tend to have better perception, which can perceive the support from others and from the organization and society (Ren and Ji, 2019; Xu et al., 2020). Moreover, psychological capital, as a positive psychological resource, has the effect of enhancing motivation and replenishing energy (Datu et al., 2016).

On the basis of correlation analyses, this study explores the mediation path between psychological capital and volunteering behavior by introducing two elements, namely, perceived social support and voluntary motivation, and by adopting an integrated approach to reveal the impact of volunteer psychological capital on volunteering behavior, and thus opening the “black box” of its underlying mechanism. It not only helps us to understand how the psychological capital of the volunteers affects the generation, maintenance, and sustainable development of volunteering behavior from the perspective of both conservation of resources theory and self-determination theory, but also helps to integrate conservation of resources theory and self-determination theory to improve the explanatory power of the mediation effect. The mediating effect analysis shows that voluntary motivation has a significant mediating effect on the psychological capital of the volunteers. Psychological capital, as a positive psychological quality or state, can awaken and stimulate individual voluntary motivation (Luthans et al., 2004). Meanwhile, according to the conservation of resources theory, psychological capital is regarded as a type of psychological resource that can help to strengthen more positive behaviors (Hobfoll et al., 2018; Xu et al., 2020). Moreover, self-determination theory posits that it plays an important role in activating, inducing, directing, and maintaining individual volunteering behavior with clearly defined voluntary service needs and goals (Ryan and Deci, 2000; Li L. et al., 2016). This is consistent with previous findings that voluntary motivation is a proximal factor whereby positive psychological capital affects individual behavior (Horowitz et al., 2001; Xu et al., 2020). Besides, the results of this study show that the indirect effect of perceived social support on the effect of psychological capital of volunteers on individual volunteering behavior is not significant, but that the psychological capital of volunteers is a direct predictor of perceived social support. This shows that there may be other variables between perceived social support and voluntary behavior. In other words, the perceived social support of volunteers may have an effect on voluntary behavior through one or several variables. Meanwhile, the reason why the psychological capital of volunteers can positively predict and comprehend social support is related to the fact that psychological capital, as a positive psychological resource, can enhance the ability of individuals to perceive or experience support and help from external environment (Hobfoll et al., 2018; Jiankun et al., 2018).

The present study also finds that the chained mediation of “perceived social support → voluntary motivation” is an important pathway, whereby the psychological capital of volunteers influences volunteering behavior. Although there have been few direct studies on the effect of perceived social support on voluntary motivation, studies on the relationship between social support and motivation show that it plays a significant positive predictive role in the effect of perceived social support on learning motivation (Bagci, 2018). Perceived social support is a cognition of individuals of being supported, cared for, and helped by family members, society, or organizations through their own networks (Wentzel, 1994), and it is a perception of objective support from the external environment (Thoits, 1983). Voluntary motivation is an inner mental process that is motivated, guided, and maintained by volunteer goals or targets (Compton et al., 2004). Self-determination theory argues that motivation is a state inherent in any individual, but not entirely an internal state. Whether this state is stimulated depends mainly on the dynamic interaction between the individuals and their environment (Ryan et al., 2009). Voluntary motivation is the result of the dynamic interaction between volunteers and their environment. Volunteers often have several needs or goals in the process of interacting with the environment, which require volunteers to effectively adjust themselves to the environment (Myers, 2006). Therefore, voluntary motivation can be regarded as a result of the dynamic interaction between volunteers and their environment. Self-determination theory posits that the formation and development of individual positive goals are inseparable from the common function of the external environment (Deci and Ryan, 2000). The establishment of new goals of the volunteers and the extent of their motivation are influenced by whether the dynamics of their goals and intentions can effectively match their perception of the external support of family, society, or an organization (Muradian Sarache and Rival, 2012). When individuals can become fully perceived of support from families, society, or voluntary organizations, their voluntary goals and intentions will be further stimulated, promoting the formation, stability, and sustainable development of volunteering behaviors.

This study reveals some of the mechanisms whereby the psychological capital of volunteers affects volunteering behavior by constructing a chained mediation model. The psychological capital of volunteers can predict volunteering behavior through the independent mediation role of voluntary motivation and the chained mediation role of perceived social support and voluntary motivation. The results regarding the independent mediating role of voluntary motivation endorse self-determination theory to some extent. Moreover, the chained intermediary role of perceived social support and voluntary motivation effectively integrates conservation of resources theory and self-determination theory, which is of great value to revealing the combined role of perceived social support and voluntary motivation in the relationship between the psychological capital and volunteering behavior of volunteers. This implies that raising the level of the psychological capital of the volunteers will stimulate their voluntary motivation and further promote volunteering behavior, as well as voluntary goals and intentions, through perceived social-support-enhancing voluntary motivation, thus promoting the formation and development of volunteering behaviors.

### Theoretical Contribution

The theoretical contributions of this study mainly include the following three aspects: firstly, the present study reveals the direct effects of psychological capital on perceived social support, voluntary motivation, and voluntary behavior. Individuals with high psychological capital tend to perceive higher levels of support from others and from organizations and society, which contributes to the formation of voluntary motivation, and which in turn translates into voluntary service behavior. This enriches the theoretical research on the effect of psychological capital. Secondly, the present study analyzes the emergence and development of volunteering behavior from the perspective of psychological capital, whereas previous studies mainly analyze it from the perspectives of physical capital, social capital, and human capital. This broadens the analytical perspective of voluntary behavior research. Thirdly, the present study creatively integrates the factors of perceived social support and voluntary motivation into the theoretical model of the influence of volunteer psychological capital on voluntary behavior. It is found that perceived social support and voluntary motivation have the chain mediating effect on the psychological capital of the volunteer to voluntary behavior, which has no report in previous studies.

### Limitations and Future Research

There were also some limitations in this study. Firstly, the present study used crosssectional methods only, so this research may be restricted by causal inferences. Therefore, we encourage the use of longitudinal experiments in future studies to draw causal inferences among psychological capital, perceived social support, voluntary motivation, and volunteering behavior. Secondly, this study only discussed how the psychological capital of volunteers predicted volunteering behavior. Previous studies revealed that role identification could play a mediating or moderating role (Callero et al., 1987; Ngan et al., 2011; Li and Zhou, 2017; Song et al., 2018), and perceived social support could be considered as a moderating variable rather than an intermediary variable (Fontanini et al., 2014; Miloseva et al., 2017). It was obvious that previous studies mostly focused on employees, whereas this study focused on volunteers. In addition, we also encourage future research to introduce moderating variables to further discuss how the psychological capital of volunteers affects volunteering behavior. Thirdly, in this study, we did not distinguish the volunteers engaging in different volunteer activities, but discussed them as a whole, which helped us to understand the volunteer group as a whole. There is no doubt that there may be some differences in psychological capital and voluntary motivation of volunteers engaging in different voluntary activities. Therefore, we encourage future research to explore the moderating role of different types of voluntary activities in the relationship between voluntary psychological capital and voluntary behavior. Last but not least, the analysis of the mediating effects in this study have shown that the direct effect of psychological capital on volunteering behavior was much stronger than the indirect effect, suggesting that more attention should be paid to changing and developing the psychological capital of volunteers, while improving the level of the psychological capital of volunteers through certain interventions that would promote volunteering behavior and its sustainable development. It was also enlightening that there might be other important variables in the process whereby volunteer psychological capital affected volunteering behavior. So we suggest that qualitative research should be applied to explore other core elements of the psychological capital of the volunteer and promote the development of volunteering behavior.

## Conclusion

The psychological capital of volunteers, perceived social support, voluntary motivation, and volunteering behavior were significantly and positively related to each other and voluntary motivation had a significant mediating effect on the psychological capital of volunteers. Moreover, perceived social support and voluntary motivation also had a chain effect on the relationship between the psychological capital and volunteering behavior of the volunteers.

## Data Availability Statement

The original contributions presented in the study are included in the article/supplementary material, further inquiries can be directed to the corresponding authors.

## Ethics Statement

The studies involving human participants were reviewed and approved by this study was carried out in accordance with academic Ethics guidelines, and the recommendations of the Committee of Social Sciences Department of Zunyi Medical University Zhuhai Campus, which also approved the study protocol. All subjects provided written informed consent in accordance with the Declaration of Helsinki. Written informed consent to participate in this study was provided by the participants' legal guardian/next of kin.

## Author Contributions

LX wrote this manuscript, as well as designed, performed, analyzed, and critically revised the research. JL, YW, and HK searched literature. All authors contributed to the article and approved the submitted version.

## Funding

This research was funded by the China Social Science Fund Project (NO: 17CGL039) and Doctoral start-up fund project of Zunyi Medical University (FB-2019-5).

## Conflict of Interest

The authors declare that the research was conducted in the absence of any commercial or financial relationships that could be construed as a potential conflict of interest.

## Publisher's Note

All claims expressed in this article are solely those of the authors and do not necessarily represent those of their affiliated organizations, or those of the publisher, the editors and the reviewers. Any product that may be evaluated in this article, or claim that may be made by its manufacturer, is not guaranteed or endorsed by the publisher.
